# The effects of house moves during early childhood on child mental health at age 9 years

**DOI:** 10.1186/1471-2458-12-583

**Published:** 2012-08-01

**Authors:** Alice R Rumbold, Lynne C Giles, Melissa J Whitrow, Emily J Steele, Christopher E Davies, Michael J Davies, Vivienne M Moore

**Affiliations:** 1 Discipline of Obstetrics & Gynaecology, The University of Adelaide, Adelaide, SA 5005, Australia; 2 Discipline of Public Health, The University of Adelaide, Adelaide, SA 5005, Australia; 3 Menzies School of Health Research, Darwin, NT, 0811, Australia

**Keywords:** Residential mobility, Child behaviour, Child development, Housing, Longitudinal studies

## Abstract

**Background:**

Residential mobility is common in families with young children; however, its impact on the social development of children is unclear. We examined associations between the number, timing and type of house moves in childhood and child behaviour problems using data from an ongoing longitudinal study.

**Methods:**

Complete data on residential mobility and child behaviour was available for 403 families. Three aspects of mobility were considered: (a) number of house moves from birth to <2 years, 2 to <5 years and 5 to 9 years; (b) lifetime number of house moves; and (c) moves associated with different housing trajectories characterized by changes in housing tenure. The primary outcomes were internalizing and externalizing behaviour problems at 9 years derived from Achenbach’s Child Behaviour Checklist. Linear regression analyses were used to investigate the effect of the housing variables on internalizing and externalizing behaviour problem scores with adjustment for a range of sociodemographic and household covariates.

**Results:**

Moving house ≥2 times before 2 years of age was associated with an increased internalizing behaviour score at age 9 years. This association remained after adjustment for sociodemographic and household factors. There was no association between increased residential mobility in other time periods and internalizing behaviour, or mobility in any period and externalizing behaviour. There was no effect of lifetime number of moves, or of an upwardly or downwardly mobile housing trajectory. However, a housing trajectory characterized by continuous rental occupancy was associated with an increased externalizing behaviour score.

**Conclusions:**

These findings may suggest that there is a sensitive period, in the first few years of life, in which exposure to increased residential mobility has a detrimental effect on mental health in later childhood.

## Background

Residential mobility varies over the life course [1] and is high in young adults and families with young children [2,3]. High rates of residential mobility have been associated with social disadvantage including poverty [1,2,4], employment instability and family breakdown [2,5,6]. While social inequalities have long been recognised as a key determinant of health [7,8], more recently, there has been acknowledgement that inequalities start before birth and accumulate across the life course [9]. The impact of residential mobility on child health and development has been the focus of increasing research attention, although results of studies in this area have been inconsistent [10].

Several cross-sectional studies report an association between frequent moves and behavioural problems such as aggression in school-age children [11,12]. In contrast, one longitudinal study found no detrimental effect of residential mobility on anxiety and aggression in this age-group [13,14]. In another longitudinal study, mobility assessed at a neighbourhood level was shown to moderate the association between parental monitoring and externalizing behaviours (e.g. acting out, aggression) [14]. Gasper and colleagues [15] used longitudinal data to examine the effects of residential mobility on delinquency and problem behaviour in adolescence, and found that the effects of mobility on behaviour were mediated by unobserved individual and family differences between mobile and non-mobile youth.

These inconsistent findings may reflect methodological differences in study design, analytical approaches, and measurement of residential mobility. The majority of past research examining the impact of mobility on child development is cross-sectional, and therefore unable to assess temporality, with varying attention paid to addressing confounding based on socioeconomic and household characteristics [10].

To our knowledge there has been no prior examination of the stage of childhood during which the impact of mobility may be greatest. House moves at certain ages may be more detrimental than moves at other ages. For example, moves occurring when the child has commenced school may have a greater impact on behaviour than at earlier ages, due to disruption of established friendships and peer groups, which are increasingly important in later childhood and early adolescence. Also important is the potential for school moves to independently contribute to the development of behaviour problems through its impact on school achievement and coping [16].

The aim of this study was to examine the association between residential mobility during childhood and child behaviour at 9 years in a prospective cohort study. Specifically, we examined associations between child behaviour and: (a) the number of house moves occurring from birth to <2 years, 2 to <5 years, and 5 to 9 years; (b) the lifetime number of house moves from birth until 9 years; and (c) moves during childhood associated with different housing trajectories characterized by changes in housing tenure.

## Methods

### Sample and study design

The Generation 1 study is a longitudinal cohort study of women and their children (n = 557) established between 1998 and 2000 in Adelaide, South Australia. The study is described in detail elsewhere [17-19]. In brief, women were recruited prior to 16 weeks gestation at a public hospital or through the private practices of three obstetricians. Eligible women were aged ≥18 years, Caucasian, and free from certain conditions known to affect fetal growth [17]. The sample of mothers was broadly representative of all women who gave birth in South Australia during the cohort’s establishment, as described previously [17]. The University of Adelaide Human Research Ethics Committee approved the study. All participants gave written informed consent, and the study procedures conformed to the principles of the Declaration of Helsinki.

Mothers and children were followed up during pregnancy (at 16 and 32 weeks), at birth and on eight occasions in early childhood (at 3, 6, 9 and 12 months, and at 2, 3.5, 5 and 9 years). Data pertaining to the children and their wider family circumstances have been collected at each study wave.

### Outcome measures

The outcomes considered were internalizing behaviour problems (withdrawn/depressed) and externalizing behaviour problems (aggressive/destructive), derived from Achenbach’s Child Behaviour Checklist (CBCL) age 6–18 years parent report form [20]. The CBCL is a well-validated 113-item instrument designed to record children’s competencies and problems as reported by their parents. In the present study, mothers rated each CBCL item as not true (0), somewhat true (1) or very true (2), as part of the interview schedule when study children were aged 9 years. Internalizing and externalizing behaviour problem scores were derived according to standard methods [20]. Analyses were based on raw scores, which are recommended for use in community based samples [21].

### Residential mobility

Three aspects of residential mobility were assessed. The effects of the number of house moves in the periods birth to <2, 2 to <5 and 5 to 9 years, were separately considered. In each of these periods, the number of house moves was classified as 0, 1, or ≥2. The effect of the lifetime (total history of) number of house moves from birth until 9 years was also considered (0, 1, or ≥2 moves).

In order to assess moves associated with different housing trajectories, a variable describing change in housing tenure between 2, 3.5 and 9 years of age was constructed. At each of these time points, housing tenure was classified as private rental, public rental, mortgage/own (including purchase of public housing), living with extended family or living in a residence rent free. Families were classified as having an upwardly mobile housing trajectory if at any time the housing tenure changed from private rental, public rental or living with extended family to mortgage/own or living in a residence rent free, and the change was sustained (i.e. no subsequent tenure changes). Families were classified as having a downwardly mobile housing trajectory if at any time the housing tenure changed from mortgage/own or living in a residence rent free to private rental, public rental, or living with extended family, and this change was sustained. The trajectory was classified as ‘mixed’ when both upward and downward housing tenure changes occurred. Families with no upward or downward housing tenure changes were classified as having a trajectory of either continuous home ownership (including families who never moved or moved between purchased properties) or continuous rental occupancy (including families who never moved or moved between rental properties). The number of house moves in each period was used as an additional check in the derivation of the measure of housing trajectories.

### Covariates

Information on potentially relevant covariates was taken from the pregnancy questionnaires and the assessments at previous study waves. Covariates considered were maternal age at birth of the study child, child sex, highest level of maternal education prior to the birth of the study child (High School (HS) not completed, HS not completed but Technical and Further Education College (TAFE) or University completed, HS completed only, HS and TAFE completed, or HS and University completed), average annual household income prior to the birth of the study child (≤$31,199, $31,200-$51,999, ≥$52,000), change in parental relationship status (separation at any time from birth to 9 years), changes in the number of children in the household (≤1, 2, 3 or ≥4 children in the household at 2 years; ≥1 additional child in the house from 2 to 3.5 years), stressful events in the family between birth and 2 years (any of the following: family court matters, restraining orders, criminal charges, deaths in the immediate family, or any other stressful events nominated by the family), and change in school for the study child between reception (the first year of school) and 9 years (0, 1, ≥2 school moves).

### Statistical analysis

The bivariate associations between housing variables, child behaviour problem scores and covariates were investigated through chi-square tests of association for categorical variables and Mann–Whitney U-tests for variables with a continuous distribution.

Linear regression analyses were used to investigate the effect of the housing variables on internalizing and externalizing behaviour problem scores at 9 years. Models were built with the following: (1) number of house moves in each time period; (2) lifetime number of house moves at 9 years; and (3) moves associated with different housing trajectories characterized by housing tenure change. To assess the effects of sociodemographic and household covariates on the fit of each model, models that adjusted for the following factors were also included in the suite of analyses: (a) maternal age, child sex and maternal education; (b) = (a) plus household income, parental relationship status, household composition, stressful events between birth and 2 years, and change in school between reception and 9 years. A potential interaction between house moves from birth to 9 years and school moves from reception to 9 years was tested in each model.

Due to the positive skew in the distribution of the CBCL responses, a square root transformation was applied to the externalizing and internalizing problem scores, and the transformed scores were used in subsequent analyses. Results from the regression analyses are presented as regression coefficients (*β*) and their 95% Confidence Intervals (CI). A P-value <0.05 was considered statistically significant. All regression analyses were conducted using the REG procedure in SAS version 9.2 (SAS Inc, Cary, NC).

## Results

At 9 years, 443 mothers and children participated in the overall follow up. Complete information on residential status and CBCL scores was collected from 403 mothers and children (72% of the original sample). The mean age of the mothers at the birth of study child was 30.3 years (standard deviation 4.9 years) (Table1). Just over two-thirds (279, 69.2%) of mothers had completed high school or had further education/training prior to the birth of the child. The mean internalizing and externalizing behaviour problem scores at 9 years are presented in Table1.

**Table 1:** Characteristics of the mothers, children and family environment in participating families (n = 403)

**Characteristics**	**N**	**%**
**Child sex female**	207	51.4
**Child age at interview, mean SD**	9.6	0.3
**Maternal age at birth, mean SD**	30.3	4.9
Maternal education^*^		
HS not completed	124	30.8
HS not completed, TAFE or Univ. completed	61	15.1
HS completed only	68	16.9
HS completed, TAFE completed	67	16.6
HS completed, Univ. completed	83	20.6
**Household income**^**†**^		
≤$31,199	119	29.5
$31,200-$51,999	117	29.0
≥$52,000	167	41.4
**Experience of stressful events in the family from birth to 2 years**	157	39.0
**Number of children in the household at 2 years**^**‡**^		
1 child	103	25.5
2 children	192	47.6
3 children	75	18.6
≥4 children	33	8.2
**Additional children in the household from 2 to 3.5 years**		
None^§^	301	74.7
≥1 child	102	25.3
**Continuously with the same partner from birth to 9 years**	310	76.9
**Housing tenure status at 9 years**		
Purchased own house	330	81.9
Public housing rental	16	4.0
Private rental	50	12.4
Living with extended family	4	1.0
Occupying a residence rent free	3	0.7
**Child behaviour checklist at 9 years**		
Internalizing behaviour problem score, mean SD	6.3	5.6
Externalizing behaviour problem score, mean SD	6.8	6.7
Transformed internalizing behaviour problem score, mean SD	2.2	1.1
Transformed externalizing behaviour problem score, mean SD	2.3	1.3

HS = High School, SD = Standard Deviation, TAFE = Technical and Further Education College, Univ. = University.

^*^Education recorded during early pregnancy interview.

^†^Income recorded during late pregnancy interview.

^‡^Includes the study child.

^§^Includes 10 families where there were fewer children in the household at 3.5 years than at 2 years.

Mothers of children not participating at the 9 year follow up were younger, less educated and had a lower household income than mothers who did participate (P < 0.05). However, there was no difference in the number of house moves recorded from birth to <2 years, or 2 to <5 years between responders and non-responders at 9 years.

By the 9 year follow up, the majority of families (330, 81.9%) had purchased their own house (including mortgaged ownership); this included two families who purchased public housing.

Measures of residential mobility are summarised in Table2. At 9 years, 163 families (40.4%) had never moved house, 91 families (22.6%) reported one house move, and 149 (37.0%) reported moving house two or more times since the birth of the study child. Ninety-eight children (24.3%) had experienced one school move since reception and 18 children (4.5%) had experienced ≥2 school moves.

**Table 2:** Summary of measures of residential mobility from birth to 9 years (n = 403)

**Characteristics**	**N**	**%**
**House moves from birth to <2 years**		
No moves	288	71.5
1 move	84	20.8
≥2 moves	31	7.7
**House moves from 2 to <5 years**		
No moves	266	66.0
1 move	92	22.8
≥2 moves	45	11.2
**House moves from 5 to 9 years**		
No moves	262	65.0
1 move	79	19.6
≥2 moves	62	15.4
**Total house moves from birth to 9 years**		
No moves	163	40.4
1 move	91	22.6
≥2 moves	149	37.0
**Housing trajectories associated with housing tenure changes at 2, 3.5 and 9 years**		
Continuous home ownership	281	69.7
Continuous rental occupancy	34	8.4
Upwardly mobile^*^	39	9.7
Downwardly mobile^†^	29	7.2
Mixed^‡^	20	5.0

^*^Includes a sustained change in housing tenure from a private rental, public rental or living with extended family to mortgage/own or living in a residence rent free.

^†^Includes a sustained change in housing tenure from mortgage/own or living in a residence rent free to private rental, public rental, or living with extended family.

^‡^Includes families who experience both upwardly and downwardly mobile housing tenure changes.

### Number of house moves in each time period

The coefficients for the linear regression models examining house moves at each time period on internalizing behaviour at 9 years are presented in Table3. Compared with the referent group of no house moves, moving house ≥2 times from birth to <2 years of age was associated with an increased internalizing behaviour score at age 9 years (β = 0.74, 95% CI 0.31-1.18). This effect was robust to adjustment for covariates that reflected sociodemographic and household characteristics, including parental relationship breakdown and family experiences of stressful events (Models 2 and 3, Table3). There was no association between internalizing behaviour scores and one house move between birth and <2 years, or the number of house moves from 2 to <5 years or 5 to 9 years.

**Table 3:** Effects of house moves on internalizing behaviour scores at age 9 years

	Model 1^*^			Model 2^*†^			Model 3^*‡^		
	**β**	**95% CI**	**P value**	**β**	**95% CI**	**P value**	**β**	**95% CI**	**P value**
**Intercept**	2.17	2.02 - 2.32	<0.0001	2.84	1.98 - 3.71	<0.0001	2.45	1.46 - 3.41	<0.0001
**House moves from birth to <2 yrs**									
No moves	ref		0.0038^§^			0.0052^§^			0.0061^§^
1 move	0.03	-0.25 - 0.31		-0.10	-0.39 - 0.20		-0.13	-0.42 - 0.16	
≥2 moves	0.74	0.31 - 1.18		0.67	0.23 - 1.11		0.65	0.20 - 1.10	
**House moves between 2 yrs and <5 yrs**									
No moves	ref		0.48^§^			0.37^§^			0.27^§^
1 move	-0.01	-0.28 - 0.27		-0.06	-0.34 - 0.22		-0.04	-0.32 - 0.23	
≥2 moves	-0.23	-0.62 - 0.15		-0.28	-0.67 - 0.11		-0.33	-0.72 - 0.07	
**House moves between 5 yrs and 9 yrs**									
No moves	ref		0.63^§^			0.65^§^			0.47^§^
1 move	0.14	-0.15 - 0.43		0.12	-0.17 - 0.41		0.19	-0.11 - 0.48	
≥2 moves	0.04	-0.28 - 0.36		-0.03	-0.36 - 0.29		0.08	-0.29 - 0.45	
**Maternal age at birth of study child**				-0.03	-0.05 - 0.00	0.022	-0.02	-0.04 - 0.01	0.20
**Study child sex female**				0.15	-0.07 - 0.37	0.17	0.15	-0.07 - 0.38	0.17
**Maternal education**									
HS not completed				0.26	-0.06 - 0.58	0.34^§^	0.37	0.00 - 0.73	0.23^§^
HS not completed, TAFE or Univ. completed				0.18	-0.19 - 0.55		0.24	-0.15 - 0.63	
HS completed only				0.15	-0.21 - 0.51		0.18	-0.19 - 0.56	
HS completed, TAFE completed				0.36	0.01 - 0.72		0.39	0.03 - 0.75	
HS completed, Univ. completed				ref			ref		
**Income**									
≤$31,199							0.11	-0.20 - 0.43	0.64^§^
$31,200-$51,999							-0.03	-0.32 - 0.26	
≥$52,000							ref		
**With same partner from birth to 9 yrs**							0.06	-0.23 - 0.34	0.70
**Stressful events between birth and 2 yrs**							0.14	-0.09 - 0.37	0.23
**Children in the household at 2 yrs**									
1 child							ref		0.17^§^
2 children							-0.10	-0.40 - 0.20	
3 children							-0.29	-0.66 - 0.09	
≥4 children							-0.47	-0.95 - 0.00	
**≥1 additional child from 2 to 3.5 yrs**							0.17	-0.11 - 0.45	0.23
**School moves from reception to 9 yrs**									
No school moves							ref		0.32^§^
1 school move							-0.20	-0.47 - 0.06	
≥2 school moves							-0.02	-0.60 - 0.56	

CI = Confidence Interval, HS = High School, TAFE = Technical and Further Education College, Univ. = University, yrs = years.

^*^R-square values are 0.032 (Model 1), 0.062 (Model 2) and 0.091 (Model 3).

^†^Includes adjustment for maternal age, child sex and maternal education.

^‡^Includes adjustment for maternal age, child sex, maternal education, household income, parental relationship status, household composition, stressful events between birth and 2 years, and change in school between reception and 9 years.

^§^P values reflect an overall test for the inclusion of the set of variables.

The results for the linear regression models examining house moves at each time period on externalizing behaviour at 9 years are presented in Table4. With a referent group of no house moves, ≥2 house moves from birth to <2 years was associated with an increased externalizing behaviour score (β = 0.53, 95% CI 0.04-1.02), as was one house move from 2 to <5 years (β = 0.32, 95% CI 0.01-0.62); however, both of these associations were attenuated and not statistically significant after adjustment for sociodemographic covariates. The frequency of house moves from 5 to 9 years did not have a significant effect on externalizing behaviour at 9 years.

**Table 4:** Effects of house moves on externalizing behaviour scores at age 9 years

	Model 1^*^			Model 2^*†^			Model 3^*‡^		
	**β**	**95% CI**	**P value**	**β**	**95% CI**	**P value**	**β**	**95% CI**	**P value**
**Intercept**	2.06	1.89 - 2.23	<0.0001	3.39	2.44 - 4.35	<0.001	3.29	2.24 - 4.33	<0.001
**House moves from birth to <2 yrs**									
No moves	ref		0.059^§^			0.28^§^			0.58^§^
1 move	0.24	-0.07 - 0.55		0.01	-0.31 - 0.33		-0.02	-0.34 - 0.30	
≥2 moves	0.53	0.04 - 1.02		0.39	-0.10 - 0.87		0.25	-0.24 - 0.73	
**House moves between 2 yrs and <5 yrs**									
No moves	ref		0.13^§^			0.40^§^			0.59^§^
1 move	0.32	0.01 - 0.62		0.19	-0.11 - 0.49		0.14	-0.17 - 0.44	
≥2 moves	0.05	-0.39 - 0.48		-0.05	-0.47 - 0.38		-0.06	-0.49 - 0.37	
**House moves between 5 yrs and 9 yrs**									
No moves	ref		0.20^§^						0.11^§^
1 move	0.30	-0.03 - 0.62		0.24	-0.08 - 0.55	0.26^§^	0.31	-0.01 - 0.63	
≥2 moves	0.05	-0.31 - 0.41		-0.06	-0.42 - 0.30		-0.06	-0.46 - 0.34	
**Maternal age at birth of study child**				-0.05	-0.07 - -0.02	<0.001	-0.03	-0.06 - 0.00	0.025
**Study child sex female**				-0.19	-0.42 - 0.05	0.12	-0.19	-0.43 - 0.05	0.12
**Maternal education**									
HS not completed				0.58	0.23 - 0.93	0.012^§^	0.46	0.06 - 0.86	0.12^§^
HS not completed, TAFE or Univ. completed				0.18	-0.23 - 0.58		0.09	-0.33 - 0.52	
HS completed only				0.14	-0.25 - 0.54		0.10	-0.31 - 0.50	
HS completed, TAFE completed				0.39	0.00 - 0.78		0.29	-0.11 - 0.68	
HS completed, Univ. completed				ref					
**Income**									
≤$31,199							0.30	-0.04 - 0.64	0.13^§^
$31,200-$51,999							-0.01	-0.32 - 0.30	
≥$52,000							ref		
**With same partner from birth to 9 yrs**							-0.34	-0.64 - 0.04	0.029
**Stressful events between birth and 2 yrs**							-0.06	-0.31 - 0.19	0.64
**Children in the household at 2 yrs**									
1 child							ref		0.56^§^
2 children							0.08	-0.24 - 0.41	
3 children							0.04	-0.36 - 0.44	
≥4 children							-0.25	-0.77 - 0.27	
**≥1 additional child from 2 to 3.5 yrs**							0.24	-0.06 - 0.54	0.12
**School moves from reception to 9 yrs**									
No school moves							ref		0.016^§^
1 school move							-0.41	-0.70 - 0.12	
≥2 school moves							0.06	-0.57 - 0.69	

CI = Confidence Interval, HS = High School, TAFE = Technical and Further Education College, Univ. = University, yrs = years.

^*^R-square values are 0.045 (Model 1), 0.113 (Model 2) and 0.162 (Model 3).

^†^Includes adjustment for maternal age, child sex and maternal education.

^‡^Includes adjustment for maternal age, child sex, maternal education, household income, parental relationship status, household composition, stressful events between birth and 2 years, and change in school between reception and 9 years.

^§^P values reflect an overall test for the inclusion of the set of variables.

### Lifetime number of house moves

We found no association between lifetime number of house moves from birth to 9 years and internalizing behaviour scores. Compared with no house moves, moving house ≥2 times from birth to 9 years was associated with an increased externalizing behaviour score (β = 0.42, 95% CI 0.14-0.70), however this association was attenuated and no longer statistically significant after adjustment for sociodemographic covariates (data not shown).

### Housing trajectories characterized by housing tenure change

Relative to a trajectory of continuous home ownership, moving connected with an upwardly mobile housing trajectory was associated with an increased internalizing behaviour score (β = 0.42, 95% CI 0.04-0.79) (data not shown) and an increased externalizing behaviour score (β = 0.68, 95% CI 0.28-1.09) (Table5), however these associations were not statistically significant after adjustment for sociodemographic and household covariates. There was no association between either a downwardly or ‘mixed’ mobile trajectory and internalizing or externalizing behaviour scores. Relative to continuous home ownership, a trajectory characterized by continuous rental occupancy was associated with an increased externalizing behaviour score (β = 1.29, 95% CI 0.86-1.72); this remained statistically significant after adjustment for sociodemographic and household covariates (Table5).

**Table 5:** Effects of different housing trajectories characterized by housing tenure change on externalizing behaviour at age 9 years

	Model 1^*^			Model 2^*†^			Model 3^*‡^		
	**β**	**95% CI**	**P value**	**β**	**95% CI**	**P value**	**β**	**95% CI**	**P value**
**Intercept**	2.09	1.95 - 2.23	<0.001	3.01	2.12 - 3.90	<0.001	2.98	1.99 - 3.97	<0.001
**Trajectories associated with housing tenure change at 2, 3.5 and 9 yrs**									
Continuous home ownership	ref		<0.001^§^			<0.001^§^			0.014^§^
Continuous rental occupancy	1.29	0.86 - 1.72		1.01	0.56 - 1.46		0.80	0.31 - 1.30	
Upwardly mobile^¶^	0.68	0.28 - 1.09		0.46	0.05 - 0.88		0.37	-0.06 - 0.80	
Downwardly mobile^**^	0.39	-0.07 - 0.86		0.29	-0.17 - 0.75		0.24	-0.25 - 0.74	
Mixed^††^	-0.01	-0.56 - 0.53		-0.13	-0.67 - 0.41		-0.19	-0.73 - 0.36	
**Maternal age at birth of study child**				-0.03	-0.06 - -0.01	0.011	-0.03	-0.06 - 0.00	0.049
**Study child sex female**				-0.18	-0.42 - 0.05	0.120	-0.18	-0.42 - 0.06	0.13
**Maternal education**									
HS not completed				0.48	0.13 - 0.82	0.027^§^	0.45	-0.06 - 0.85	0.08^§^
HS not completed, TAFE or Univ. completed				0.11	-0.29 - 0.51		0.08	-0.34 - 0.50	
HS completed only				0.11	-0.27 - 0.50		0.08	-0.31 - 0.48	
HS completed, TAFE completed				0.43	0.05 - 0.81		0.35	-0.04 - 0.74	
HS completed, Univ. completed				ref					
**Income**									
≤$31,199							0.20	-0.16 - 0.55	0.48^§^
$31,200-$51,999							0.02	-0.29 - 0.33	
≥$52,000							ref		
**With same partner from birth to 9 yrs**							-0.20	-0.51 - 0.11	0.20
**Stressful events between birth and 2 yrs**							-0.08	-0.32 - 0.17	0.53
**Children in the household at 2 yrs**									
1 child							ref		0.46^§^
2 children							0.14	-0.18 - 0.46	
3 children							0.05	-0.35 - 0.45	
≥4 children							-0.21	-0.73 - 0.30	
**1 or more additional child from 2 yrs to 3.5 yrs**							0.24	-0.06 - 0.54	0.11
**School moves from reception to 9 yrs**									
No school moves							ref		0.047^§^
1 school move							-0.34	-0.62 - -0.07	
≥2 school moves							0.002	-0.58 - 0.58	

CI = Confidence Interval, HS = High School, TAFE = Technical and Further Education College, Univ. = University, yrs = years.

^*^R-square values are 0.099 (Model 1), 0.145 (Model 2) and 0.173 (Model 3).

^†^Includes adjustment for maternal age, child sex and maternal education.

^‡^Includes adjustment for maternal age, child sex, maternal education, household income, parental relationship status, household composition, stressful events between birth and 2 years, and change in school between reception and 9 years.

^§^P values reflect an overall test for the inclusion of the set of variables.

^¶^Includes a sustained change in housing tenure from a private rental, public rental or living with extended family to mortgage/own or living in a residence rent free.

^**^Includes a sustained change in housing tenure from mortgage/own or living in a residence rent free to private rental, public rental, or living with extended family.

^††^Includes families who experience both upwardly and downwardly mobile housing tenure changes.

The interaction between house moves and school moves was not significant in any of the models in Tables 3, 4 or 5.

## Discussion

In this study, increased residential mobility in early life (before age 2 years), was associated with increased internalizing behaviour problems in children at age 9 years. The association was robust to adjustment for maternal demographic characteristics, the child’s sex, household characteristics, family experiences of stressful life events and changes in family composition. There was no effect of increased mobility after 2 years and there appeared to be no cumulative effect of number of house moves up to 9 years. These findings suggest that there is a sensitive period in early life in which increased residential mobility, even in the absence of instability in other aspects of the home environment, impacts adversely on mental health in later childhood.

In contrast with our findings, other longitudinal studies have found no independent effect of residential mobility on child behavioural development [13,14]. This inconsistency may be explained by differences in the measurement of mobility. For example, Verropoulou et al. [13] assessed whether the child had ever moved home since birth, and separately, the impact of any move associated with a change in family status. Beyers et al. [14] measured a different construct of mobility based on the proportion of renter-occupied households and householders who had lived in the neighbourhood for less than 5 years. In this study, we quantified the number of house moves experienced by individual families and were able to examine the impact of moves at specific periods in the child’s life. As a result, our study may represent a more sensitive assessment of mobility across childhood than earlier longitudinal studies.

The mechanisms through which increased residential mobility in early life may result in the emergence of behavioural problems in later childhood are unclear. Maltreatment in infancy and early childhood has been associated with difficulties in impulse control and modulation of responses at age 4 years [22], and trajectories of anxiety/depression and attention problems from age 4 through 10 years [23]. The possible neuro-biological pathways linking early adversity to behavioural problems over the course of childhood have not been well-studied in humans, although brain plasticity in the early years is now well-established [24,25] . Early deprivation has been studied in a range of animal species, with evidence of permanent alteration in stress regulation processes [26,27].

Moving house is considered a stressful life event [28]. While the causal mechanism linking mobility in early childhood to later behaviour is conjectural, we suggest that moving disrupts family routines, and can be especially stressful for all family members if the move is not voluntary. Infants from as young as 7 months have the ability to appraise situations as stressful, and from 12 months use social referencing (cues from reactions of parents and siblings) to assess events and respond emotionally or behaviourally [29]. Therefore, young children exposed to frequent family upheaval may experience considerable stress, while not having the language skills to fully understand what is happening or to have a sense of threat alleviated. Further research investigating possible underlying causal pathways is required.

High levels of mobility can be a marker of a complex range of circumstances, including instability in other aspects of life and disadvantage, and are likely to be unevenly distributed across the population. We adjusted for a range of socioeconomic indicators, stressful events in the family, and relationship breakdown, which represents the most potent potential confounder due to it being the most common reason for a ‘non-aspirational’ house move [3]. The association was robust to adjustment for these variables. Although we cannot rule out that the association is due to residual confounding from other unmeasured factors, these would be expected to have an at most minor influence on the observed associations [30,31].

We did not find any association between moves linked to either an upwardly or downwardly mobile housing trajectory, and either of the outcomes, after controlling for sociodemographic factors. However, most families (78%) in this study were classified as having a housing trajectory of either continuous home ownership or rental occupancy, so there was limited statistical power to detect any effect of other trajectories on behaviour problems. Relative to continuous home ownership, continuous rental occupancy between 2 and 9 years was positively associated with externalizing behaviour; this association was attenuated but remained statistically significant after adjustment for sociodemographic factors. The reason for this association is unclear. Higher mobility has been associated with private rental tenure [32], however in this study, the frequency of house moves was not higher in families who continuously rented than in other families. This is consistent with our lack of effect of house moves in any period on externalizing behaviour. It is possible that continuously renting may correlate with other unmeasured markers of disadvantage associated with behaviour, for example, social isolation [33]. It is possible that our measure of housing trajectories did not capture all changes in housing tenure status across childhood. However, the number of house moves in each interval was also used in the derivation of the housing trajectories. The proportion of misclassified trajectories, and any effect on their association with child behaviour, is likely to be small. Further investigation of the common circumstances occurring in families who continuously rent is required.

The strengths of this study include the prospective measurement of housing exposures at several periods in childhood, and the application of widely used behavioural assessments with evidence of reliability and validity in this age-group [20]. Furthermore, although directly comparable official statistics concerning house moves among families with young children are not available, data from the Australian 2007–8 Survey of Income and Housing showed that among households with dependent children (i.e. all ages), 47% had moved at least once in the previous 5 years [34]. This is consistent with our data: in the five year interval spanning child age 5 to 9 years, 35% of study children had moved house at least once, and 60% of study children had moved at least once between birth and age 9 years.

The study has the limitation that complete data on housing and child behaviour at the 9 year follow up were available for only 72% of the original cohort. It is possible that the most mobile families did not participate in this follow-up, as mobility is likely to affect both inclusion and ongoing participation in research. As a result, our observed effects may be conservative estimates. It is also possible that inclusion of measures of child behaviour prior to any moves could have changed the results and their interpretation, if families with children with difficult temperaments and/or later high internalizing behaviour problem scores were more likely to move. The study also has the limitation that only a low proportion of the variance in behaviour is explained by each of the models, suggesting that not all relevant factors were included.

## Conclusion

This study has shown that detrimental effects of increased residential mobility on child behaviour may be established in the first few years of life. Our findings highlight the need for further studies to delineate possible underlying mechanisms and for future studies to measure mobility at multiple time-points in the life course. They have particular significance in light of evidence that Australia has very high housing costs relative to other markets [35], and that problems with housing affordability, a determinant of mobility, are particularly prevalent among young couples with children [36]. Our findings support efforts to address early disadvantage by targeting the broader structural factors that affect a child’s early life environment, including strategies that promote secure and positive housing experiences.

## Abbreviations

CBCL, Child Behaviour Checklist; CI, Confidence Interval; HS, High School; TAFE: Technical and Further Education College; Univ, University; Yrs, Years.

## Competing interests

The authors declare that they have no competing interests.

## Authors’ contributions

VMM and MJD established the cohort on which this study is based, ARR played a primary role in the interpretation of study results and wrote the first draft of the manuscript. CED undertook the statistical analyses under the supervision of LCG. All authors contributed to the writing and critical review of the manuscript. All authors read and approved the final manuscript.

## Pre-publication history

The pre-publication history for this paper can be accessed here:

http://www.biomedcentral.com/1471-2458/12/583/prepub
